# Evaluation of the Potential Targets of Shenxian–Shengmai Oral Liquid in Treating Sick Sinus Syndrome Based on Network Pharmacology and Molecular Docking

**DOI:** 10.1002/fsn3.4587

**Published:** 2024-11-12

**Authors:** Ping Hou, Heng Zhang, Dong‐Yu Min, Jie Wu, Chen Chen, Jie Wang, Yong‐Ping Lu, Ying‐Jia Yao, Ling‐Kang Li, Yue Liu

**Affiliations:** ^1^ Graduate School Liaoning University of Traditional Chinese Medicine Shenyang Liaoning China; ^2^ Department of Rehabilitation Medicine Shandong Provincial Hospital Affiliated to Shandong First Medical University Jinan Shandong China; ^3^ Experimental Center of Traditional Chinese Medicine Affiliated Hospital of Liaoning University of Traditional Chinese Medicine Shenyang Liaoning China; ^4^ School of Public Health Shenyang Medical College Shenyang Liaoning China; ^5^ School of Traditional Chinese Medicine Shenyang Medical College Shenyang Liaoning China; ^6^ Department of NHC Key Laboratory of Reproductive Health and Medical Genetics Liaoning Research Institute of Family Planning (The Affiliated Reproductive Hospital of China Medical University) Shenyang Liaoning China; ^7^ College of Life and Health Sciences Northeastern University Shenyang Liaoning China

**Keywords:** BMP4, molecular docking, network pharmacology, potassium ion channel, sick sinus syndrome

## Abstract

Shenxian–Shengmai (SXSM) is a Chinese patent medicine used in the treatment of sick sinus syndrome (SSS). However, its active chemical compounds and the underlying molecular mechanisms remain unclear. In this study, we researched the underlying mechanisms of SXSM in treating SSS. We conducted network analysis and molecular docking to identify the small molecules and core targets responsible for the therapeutic efficacy of SXSM on SSS. In vitro experiments were performed to verify the potential therapeutic mechanism. Network pharmacological analysis identified 17 core targets. Among these, BMP4, KCNH2, KCNMA1, and KCNQ1 were identified to be involved in various biological processes, such as the formation and regulation of the cardiac pacemaking system and potassium ion transmembrane transport. The experimental analysis revealed that SXSM could upregulate the expression of the *Bmp4/Tbx3/Hcn4* pathway and the expression of *Kcnh2*, *Kcnma1*, and *Kcnq1* channels, which protected and improved the pacemaking function of pacemaker cells (P cells) and increased the heart rate. These findings provide a scientific basis in the study of the mechanism of traditional Chinese medicine in the treatment of SSS.

## Graphical abstract

Shenxian–Shengmai (SXSM) is the first Chinese patent medicine that has been specially designed for sick sinus syndrome. It could increase the expression of the *Bmp4*/*Tbx3*/*Hcn4* pathway and *Kcnh2*, *Kcnq1*, *Kcnma1* potassium channels. These regulations may shorten the action potential duration of P cells and increase the heart rate.

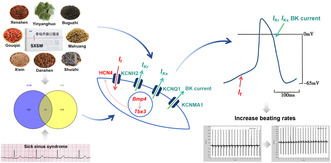

## 1 Introduction

Sick sinus syndrome (SSS) refers to a variety of arrhythmias, such as sinus bradycardia, sinus arrest, and sinoatrial block, as the main manifestations caused by the disorder of the pacemaking and/or conduction function of the sinus node tissues. These disorders reduce the heart rate, which may result in major cardiovascular events, thromboembolism, inadequate heart rate response to stress or exercise (chronotropic incompetence), or any other symptom requiring pacemaker implantation (De Ponti et al. 2018). SSS is a commonly reported arrhythmia and, frequently, the cause for cardiac consultation. With the aging of the population, the number of newly diagnosed cases of SSS in the United States is projected to increase from 78,000 in 2012 to 172,000 by 2060 (Chen and Wu 2023). The clinical manifestations of SSS include lightheadedness, palpitation, dyspnea on exertion, chest tightness, and chronic fatigue. In severe cases, consciousness disturbance, syncope, and even death have been reported (Makita et al. 2014). Because SSS refers to a series of diseases, it involves several different pathophysiological mechanisms, including degenerative fibrosis, inherited primary arrhythmia syndromes caused by gene mutations, ion channel diseases, atrial myopathies such as amyloidosis and connective tissue diseases, and secondary metabolic disorders, among others. Therefore, the etiological and effective treatment of SSS is of great significance. The goal of treatment in SSS is to increase the heart rate, normalize cardiac output, and maintain the perfusion of the brain and other terminal organs to meet physiological needs. For patients with symptoms directly attributable to SSS, cardiac pacing can increase the heart rate and improve the related symptoms. However, pacemaker therapy cannot be fully popularized because of the high medical cost involved. At the same time, although pacemaker implantation is a relatively low‐risk cardiac procedure, it may be challenged by procedural complications and death directly related to the implantation; also, the implanted battery and leads have been reported to have long‐term management implications (Strathmore et al. 2017; Lee et al. 2018; Montgomery and Ellis 2018; Liu et al. 2020). In addition, several studies have estimated that about 30% of pacemakers have been implanted in other indications besides Class I and IIa. In this group of patients, the continuous need for pacing remains debatable, and how the pacing treatment can be interrupted remains unclear. In patients unwilling to undergo pacemaker implantation and in those who are not candidates for permanent pacing, oral medicine can be considered for the treatment of symptomatic SSS (Kusumoto et al. 2019). Currently, no mainstream medicine is deemed suitable for the long‐term treatment of SSS, primarily owing to the limitation of the dosage forms and the side effects (Li, Zhang, and Shuai 2014; Greene et al. 2018). Therefore, for patients receiving conservative treatment, scientifically proven safe and effective traditional Chinese medicine modalities are urgently needed.

Shenxian–Shengmai (SXSM) is the first Chinese patent medicine that has been specially designed for SSS with precise and effective formulation and quality. It is also known to exert curative effects on SSS, with minimal side effects (Hu, Chen, and Hua 2012; Hu et al. 2016; Liu et al. 2018). It is composed of 8 Chinese medicinal materials, namely Ginseng Radix et Rhizoma (Renshen), Epimedii Folium (Yinyanghuo), Psoraleae Fructus (Buguzhi), Lycii Fructus (Gouqizi), Ephedrae Herba (Mahuang), Asari Radix et Rhizoma (Xixin), Salviae Miltiorrhizae Radix et Rhizoma (Danshen) (Table 1), and Hirudo (Shuizhi). However, it involves numerous chemical compounds and a complicated metabolic process, which makes it difficult to explore its therapeutic targets and the action mechanisms against SSS.

**TABLE 1:** Ingredients of SXSM used with accepted names.

Latin name of herbal drugs	Chinese name	Latin name of medicinal plants	Family	Medication part
Ginseng Radix et Rhizoma	Renshen	*Panax ginseng* C. A. Mey.	Araliaceae	Root
Epimedii Folium	Yinyanghuo	*Epimedium brevicornu* Maxim.	Berberidaceae	Leaf
Psoraleae Fructus	Buguzhi	*Psoralea corylifolia* L.	Leguminosae	Berry
Lycii Fructus	Gouqizi	*Lycium barbarum* L.	Solanaceae	Berry
Ephedrae Herba	Mahuang	*Ephedra intermedia* Schrenk et C. A. Mey.	Ephedraceae	Stem
Asari Radix et Rhizoma	Xixin	*Asarum sieboldii* Miq.	Aristolochiaceae	Root
Salviae Miltiorrhizae Radix et Rhizoma	Danshen	*Salvia miltiorrhiza* Bge.	Lamiaceae	Root

Traditional Chinese medicine prescription contains several natural chemical ingredients that are a valuable source of Chinese original drug research. The treatment of complex diseases needs to develop from a single target to a comprehensive and systematic network regulation, which is the advantage of traditional Chinese medicine treatment. In recent years, improvement in the understanding of the relationship between medicine targets and biological networks has assisted in the identification of several drugs with multiple targets that can interact with each other from the perspective of biological regulatory networks. Consequently, the concept of “network pharmacology” has been proposed. The present study employed the network pharmacology method as a comprehensive and systematic analysis tool and aided in the identification of active compounds present in SXSM, the exploration of the associated targets, and the analysis of the underlying mechanism involved in the treatment of SSS at the molecular level. Following this, molecular docking and in vitro experiments were employed to explain the main underlying mechanism.

## 2 Materials and Methods

### 2.1 Network Pharmacology Analysis

#### 2.1.1 Collection of Compounds and Targets of SXSM

Six herbs of SXSM (namely, Ginseng Radix et Rhizoma, Epimedii Folium, Lycii Fructus, Ephedrae Herba, Asari Radix et Rhizoma, and Salviae Miltiorrhizae Radix et Rhizoma) were searched using the TCMSP (https://old.tcmsp‐e.com/tcmsp.php). The active compounds were screened according to oral availability (OB) ≥ 30% and drug‐like properties (DL) ≥ 0.18 (Ru et al. 2014). Data of the compounds of Psoraleae Fructus and Hirudo were collected from the literature, and the active compounds were screened by GI absorption, Lipinski, Ghose, Veber, Egan, and Muegge on the SwissADME database (http://www.swissadme.ch/). The SMILES molecular format of the compounds was uploaded to the SwissTargetPrediction database (http://www.swissadme.ch/) to investigate the targets (Gfeller et al. 2014).

#### 2.1.2 Collection of the Targets of SSS

The keywords “sick sinus syndrome,” “sinus node dysfunction,” “bradyarrhythmia,” and “bradycardia” were searched in OMIM (https://omim.org/), DrugBank (https://go.drugbank.com/), and DisGeNET (https://www.disgenet.org/) databases. The results obtained were combined with those from the literature.

#### 2.1.3 Protein–Protein Interaction (PPI) Network Construction

The potential targets of SXSM and SSS‐related targets were intersected to obtain the common targets with reference to the online Venn 2.1 diagram (http://bioinformatics.psb.ugent.be/webtools/Venn/). The protein name of the common targets was uploaded to the String database (https://cn.string‐db.org/), choose “homo sapiens” for “organisms”, choose “0.4” for “minimum required interaction score” and hide disconnected nodes in the network to obtain the PPI network information. The analysis results were saved in the “TSV” format. The results were input into the Cytascape 3.7.2 software, and the network analyzer function was selected under the “tools” option to analyze the topological parameters of each node in the network (Shannon et al. 2003; Liu et al. 2023). The core targets were screened according to the condition such that the degree, betweenness centrality, and closeness centrality attribute values were greater than the median values.

#### 2.1.4 GO Enrichment and KEGG Pathway Analysis

The core targets were further analyzed with reference to the DAVID 6.8 database (https://David.ncifcrf.gov/) for gene ontology (GO) and Kyoto Encyclopedia of Genes and Genomes (KEGG) enrichment analyses (Huang Da, Sherman, and Lempicki 2009a, 2009b). We selected biological process (BP), cellular component (CC), and molecular function (MF) for GO enrichment analyses and performed KEGG enrichment analyses to target the gene pathway. Rank from small to large according to the *p* value, and the top 20 results (*p* < 0.05) were added to the bar charts.

### 2.2 Molecular Docking

The target protein names of KCNH2, KCNQ1, and KCNMA1 were entered in the RCSB PDB database to search for and select the appropriate protein crystal structure and then saved in the pdb format. The target protein name of BMP4 was entered in AlphaFold Protein Structure Database to download the pdb file of the predicted structure. The information about target proteins is shown in Table S1. The PyMOL 2.4.2 software was used to remove water and phosphate from the protein molecules. The PubChem database was employed to download the compound structures, and Chem3D software was used to create the corresponding 3D structures. AutoDockTools 1.5.6 software was used to convert the pdb format of the compound and the target protein file into the pdbqt format, and the active pockets were searched. The Vina script was used to calculate the molecular binding energy and perform molecular docking (Trott and Olson 2010). Finally, the visual analysis was performed with PyMOL 2.4.2 and Discovery Studio 2021.

### 2.3 In Vitro Experiments

#### 2.3.1 Reagents

SXSM was purchased from Buchang Pharmaceutical Co. Ltd. (Shandong, China, CFDA national drug standard no. WS_3_‐065 (Z‐010)‐2003 (Z); CFDA approval no. Z20080183; batch no. 1010466770). All Chinese medicinal materials of SXSM were purchased from Anhui Guangyintang Chinese Medicine Co. Ltd. and AnguoKangshenghua Medicine Ltd. (Baoding, China). The cardiomyocyte culture medium, CardioExcyte 96‐electrode plate, and the cardiomyocyte plating solution were obtained from Help Stem Cell Innovation (Nanjing, China). Dulbecco's Modified Eagle's medium (DMEM)/F12 and penicillin/streptomycin were obtained from Hyclone (USA), and fetal bovine serum was obtained from Sijiqing Biotechnology (Beijing, China). Phosphate‐buffered saline (PBS) was purchased from Solarbio Biotechnology (Beijing, China). siRNA sequences were designed and synthesized from Ribo Bio (Guangzhou, China). Lipofectamine RNAiMAX reagent and Trizol reagent were obtained from Thermo Fisher Scientific (USA). The TaKaRa PrimeScript RT Master Mix reagent and TaKaRa TB Green *Premix Ex Taq* II reagent were obtained from Takara Bio (Japan).

#### 2.3.2 Cell Culture

The HL‐1 cell line was obtained from Procell Life Science & Technology Co. Ltd. (Hubei, China). These cells were seeded onto cell culture dishes and cultured in the DMEM/F12 medium supplemented with 10% fetal bovine serum and 1% penicillin/streptomycin. The medium was refreshed every 48 h. These cells were maintained in an incubator at 37°C under a 5% CO_2_ atmosphere. The cells were further categorized into the control, si‐NC, model, SXSM, and si‐NC + SXSM groups. The cells in the si‐NC and si‐NC + SXSM groups were transfected with nonsense siRNA. The cells in the model and SXSM groups were transfected with the siRNA targets on *Bmp4*, *Kcnh2*, *Kcnq1*, and *Kcnma1*, respectively. At 24 h of transfection, the cells in the SXSM and si‐NC + SXSM groups were treated with 1 mL/L SXSM. The appropriate concentration used was inferred from the results of CCK‐8 cell viability determination, as suggested by Zhang et al. (2021). The hiPSC‐AMs were obtained from Help Stem Cell Innovation (Nanjing, China) and used to simulate P cells and cultured in the cardiomyocyte culture medium that was refreshed every 48 h (Mandel et al. 2012). The cells were stored in an incubator at 37°C under a 5% CO_2_ atmosphere. We assigned the cells to the control, si‐NC, model, and SXSM groups. The si‐NC cells were transfected with nonsense siRNA, while the cells in the model and SXSM groups were transfected with the siRNA targets of *BMP4*, *KCNH2*, *KCNQ1*, and *KCNMA1*, respectively. Finally, the cells in the SXSM group were treated with 1 mL/L SXSM after 24 h of siRNA transfection.

#### 2.3.3 siRNA Transfection

Lipofectamine RNAiMAX reagent was used to transfect the siRNA‐carrying plasmid into HL‐1 cells and hiPSC‐AMs. We employed real‐time quantitative PCR (RT‐qPCR) to detect the interference efficiency of each siRNA sequence and obtain the siRNA sequence with better interference efficiency. The target sequences are presented in Table 2.

**TABLE 2:** siRNA target sequence.

Cell	siRNA	Target sequence
HL‐1	si‐*Bmp4*	CCAGACTAGTCCATCACAA
	si‐*Kcnh2*	GGACATCCTTATCAATTTC
	si‐*Kcnq1*	CCATCATTGACCTCATCGT
	si‐*Kcnma1*	CCTGTTAGATGGTCCCTTT
hiPSC‐AMs	si‐*BMP4*	CGAGACTGGTCCACCACAA
	si‐*KCNH2*	CCGTAAGTTCATCATCGCC
	si‐*KCNQ1*	GCCAAGAAGAAATTCCAGC
	si‐*KCNMA1*	GGACCAAGACGATGATGAT

#### 2.3.4 Real‐Time Quantitative PCR

The total RNA of HL‐1 cells was extracted with the Trizol reagent. Purified RNA was reverse‐transcribed by the TaKaRa PrimeScript RT Master Mix reagent under the following reaction conditions: reverse transcription reaction at 37°C for 15 min, followed by an inactivation reaction of reverse transcriptase at 85°C for 5 s. RT‐qPCR was performed using the TaKaRa TB Green *Premix Ex Taq* II reagent on the 7500 Real‐Time PCR System (Applied Biosystems; Thermo Fisher Scientific). The RT‐qPCR amplification procedure was as follows: stage 1: pre‐denaturation at 95°C for 30 s; Stage 2: denaturation at 95°C for 5 s; annealing at 60°C for 34 s; and repeat for 40 cycles. β‐actin was used for endogenous control, and the relative expression of mRNA was quantified using the 2^−△△CT^ method. The primer sequences used in this study are listed in Table 3.

**TABLE 3:** RT‐qPCR primer sequence.

Target gene	Forward primer (5′–3′)	Reverse primer (5′–3′)
*Bmp4*	ATTCCTGGTAACCGAATGCTG	CCGGTCTCAGGTATCAAACTAGC
*Tbx3*	AGCGGGGTACAGAGATGGTC	TTGGCCTTTTTATCCAGTCCAG
*Hcn4*	GACCTGACGATGCTGTTGCT	CGGAAGTTGAGGACCAAGTCA
*Kcnh2*	CCTGCGATTTCCTGCATGG	AGAGGCGATCTCCACTTTGC
*Kcnma1*	ATCCTCACCCTAATACGGACC	GGACCATCTAACAGGGCTAAC
*Kcnq1*	CAGTGTCCTGTCCACTATTGAGC	ACCAGAGGCGGACCACATAT

#### 2.3.5 Measurement of Beating Rates and FPD

hiPSC‐AMs were seeded on the CardioExcyte 96‐electrode plate coated with the cardiomyocyte plating solution at the density of 5 × 10^4^ cells/well and maintained in the cardiomyocyte culture medium. The medium was refreshed every 48 h and cultured continuously for 12 days until hiPSC‐AMs reached a stable state. At 48 h of siRNA transfection, the beating rates and FPD of hiPSC‐AMs in each group were analyzed by the CardioExcyte 96 system.

#### 2.3.6 Data Analyses

All experiments were repeated thrice. Statistical analyses were performed with SPSS 26.0. The results obtained are presented as the mean ± SEM, and statistical comparisons were performed using one‐way analysis of variance (ANOVA). *p* < 0.05 was considered to indicate statistical significance.

## 3 Results

### 3.1 PPI Network for Common Targets of SXSM and SSS

**FIGURE 1:**
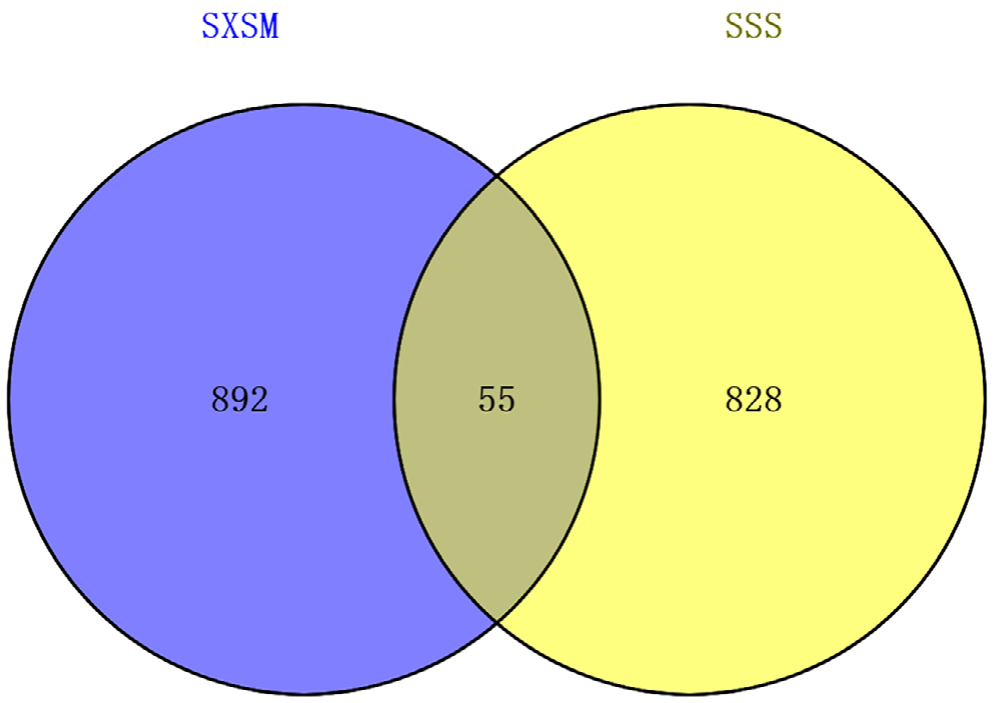
Venn diagram of the potential targets of SXSM and the targets of SSS.

**FIGURE 2:**
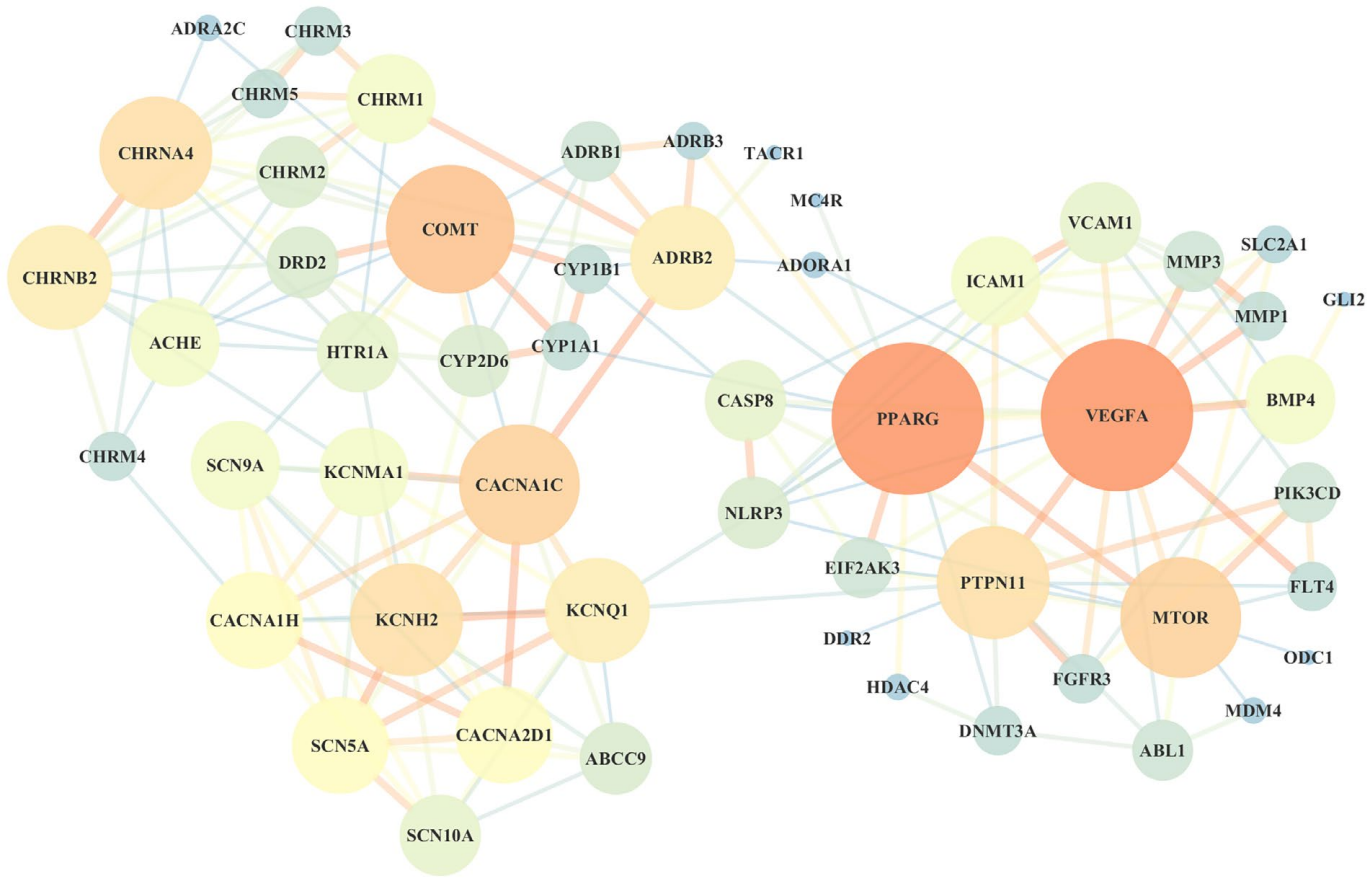
The PPI network of the potential targets of SXSM in SSS treatment.

The present study identified 947 potential targets of SXSM and 883 targets of SSS. A total of 55 common targets were identified (Figure 1). The PPI network is depicted in Figure 2. The layout is in accordance with the area and node color. The nodes represent the potential targets. The bigger and redder the nodes, the relatively larger are the degrees. The wider the edge between nodes, the greater the intensity of the red color and the stronger the interaction between the nodes. According to the topological parameters, 17 core targets were screened out, which included PPARG, COMT, MTOR, CACNA1C, KCNH2, BMP4, KCNQ1, KCNMA1, PTPN11, ADRB2, CACNA2D1, CACNA1H, CHRM1, ICAM1, VCAM1, CASP8, and CYP2D6.

### 3.2 Compound–Target Network

**FIGURE 3:**
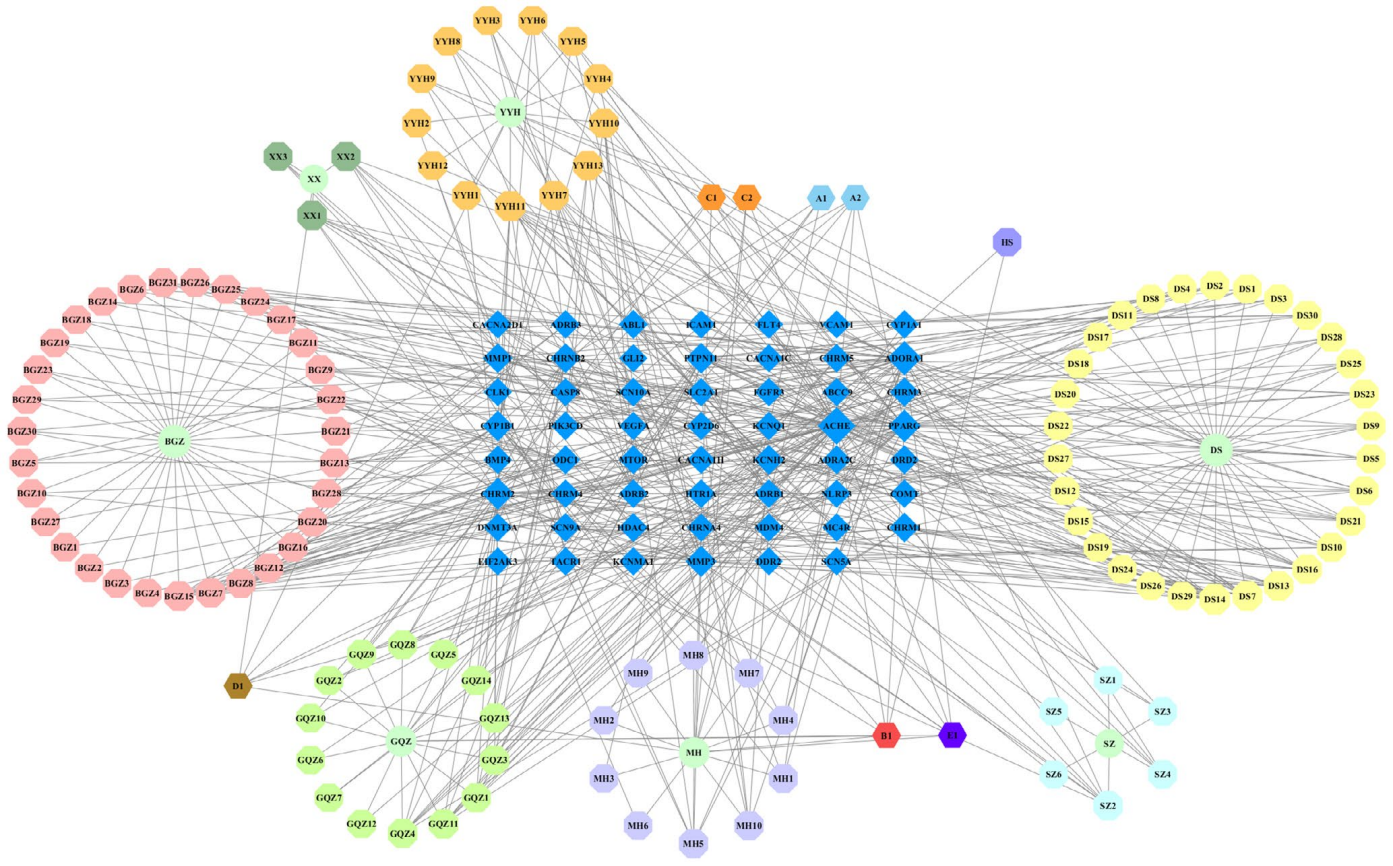
The compound‐target network of SXSM in SSS treatment.

Cytoscape 3.7.2 was used to construct the compound‐target network of SXSM (Figure 3). The layout is in accordance with the shape and area of the nodes. The ellipse nodes represent herbs, the octagon represents active compounds, the hexagon nodes represent common active compounds of different herbs, and the diamond nodes represent the potential targets of SXSM. The larger the node, the closer is the connection with other nodes.

### 3.3 GO Enrichment Analysis

**FIGURE 4:**
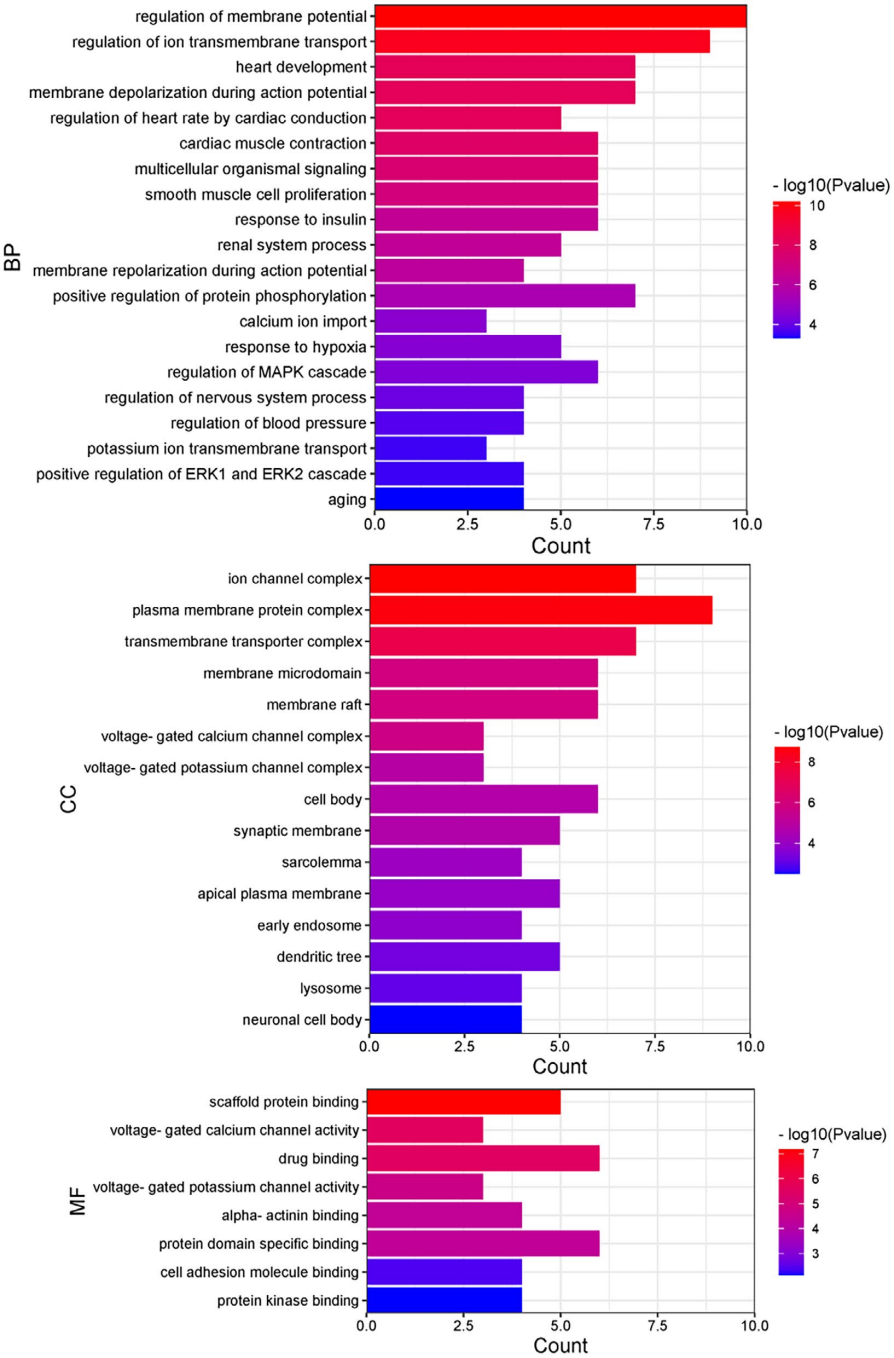
The GO enrichment analysis for 17 core targets, including BP, CC, and MF.

Furthermore, 17 core targets were analyzed using the DAVID database for GO enrichment, and 81 GO entries were obtained (*p* < 0.05), which included 62 BP terms, 15 CC terms, and eight MF terms (Figure 4). BP related to SSS was mainly concentrated in the regulation of membrane potential, regulation of ion transmembrane transport, heart development, calcium ion import, potassium ion transmembrane transport, and aging. CC mainly involved the ion channel complex, plasma membrane protein complex, membrane raft, transmembrane transporter complex, voltage‐gated calcium channel complex, voltage‐gated potassium channel complex, synaptic membrane, sarcolemma, apical plasma membrane, and cell surface. MF mainly included scaffold protein binding, voltage‐gated calcium channel activity, drug binding, voltage‐gated potassium channel activity, alpha‐actinin binding, and protein domain‐specific binding.

### 3.4 KEGG Pathway Analysis

**FIGURE 5:**
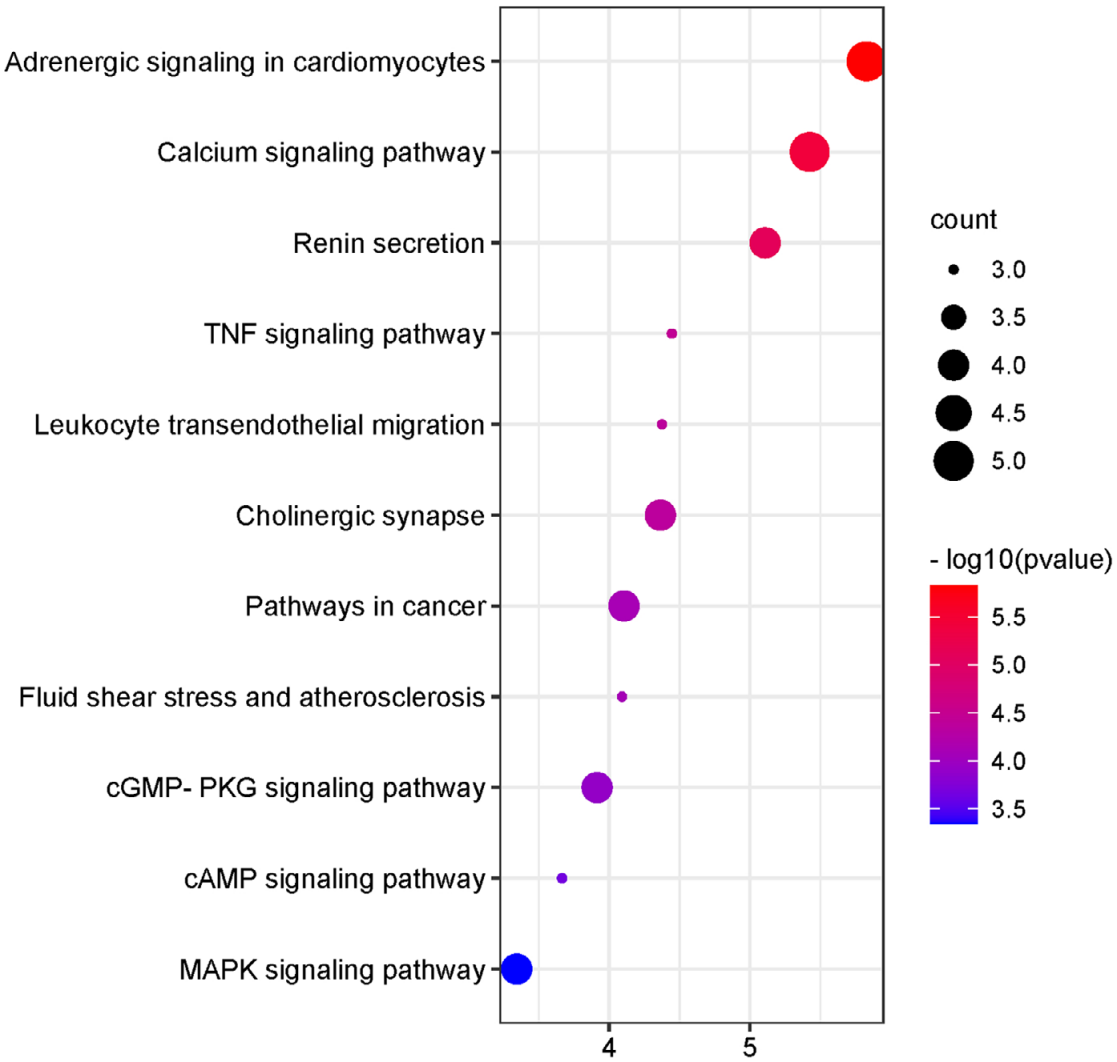
The KEGG pathway analysis of 17 core targets.

The DAVID database was used to analyze 17 core targets for the KEGG pathway analysis, and 11 pathways were obtained (*p* < 0.05; Figure 5). Among these, the targets closely related to SSS were mainly concentrated in adrenergic signaling in cardiomyocytes, calcium‐signaling pathway, renin secretion, TNF‐signaling pathway, cGMP‐PKG‐signaling pathway, and cAMP‐signaling pathway.

### 3.5 Molecular Docking

**FIGURE 6:**
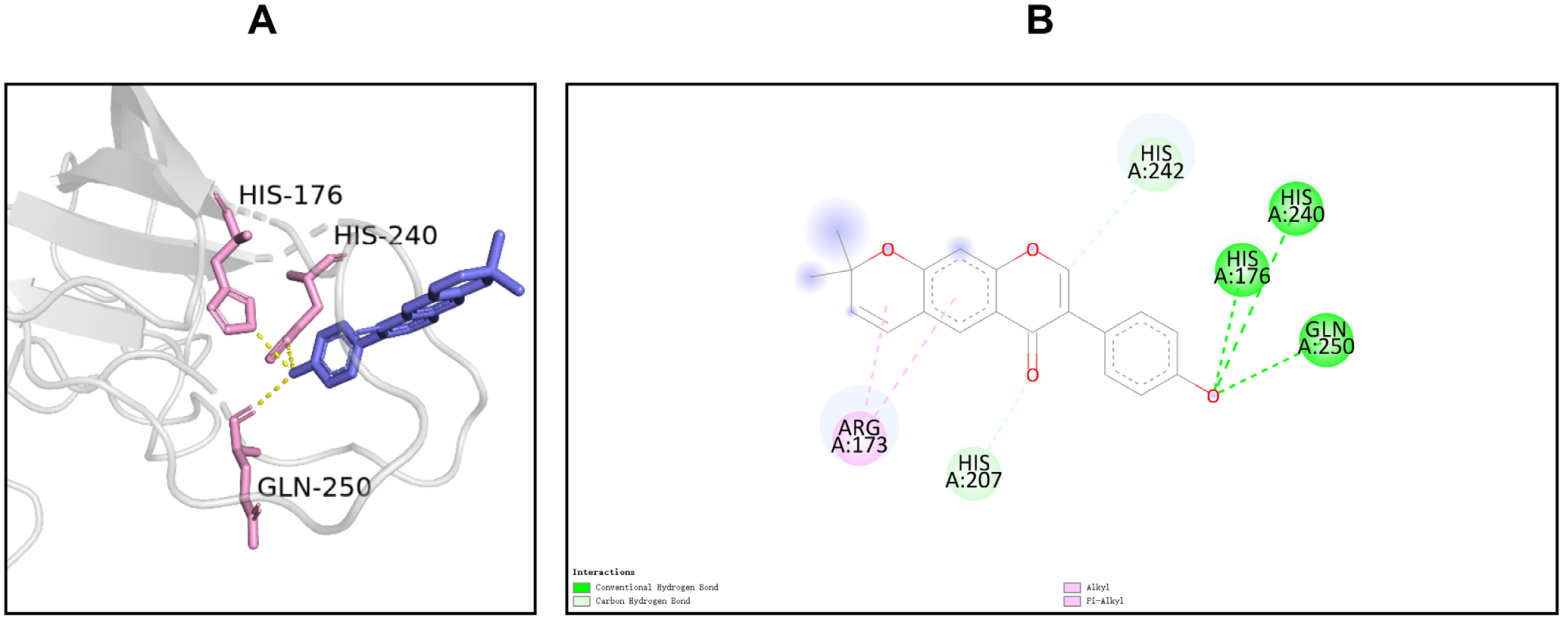
The binding mode of erythrinin A at the binding site of BMP4. (A) Schematic diagram of the 3D mode. (B) Schematic diagram of the 2D mode. BMP4 forms a hydrogen bond with erythrinin A through its His‐240, His‐176, and Gln‐250. Meanwhile, His‐242 and erythrinin A formed a weak hydrogen bond with the Pi bond as a donor; His‐207 and erythrinin A formed C–H interaction, and Arg‐173 and erythrinin A formed alkyl interaction and PI.

**FIGURE 7:**
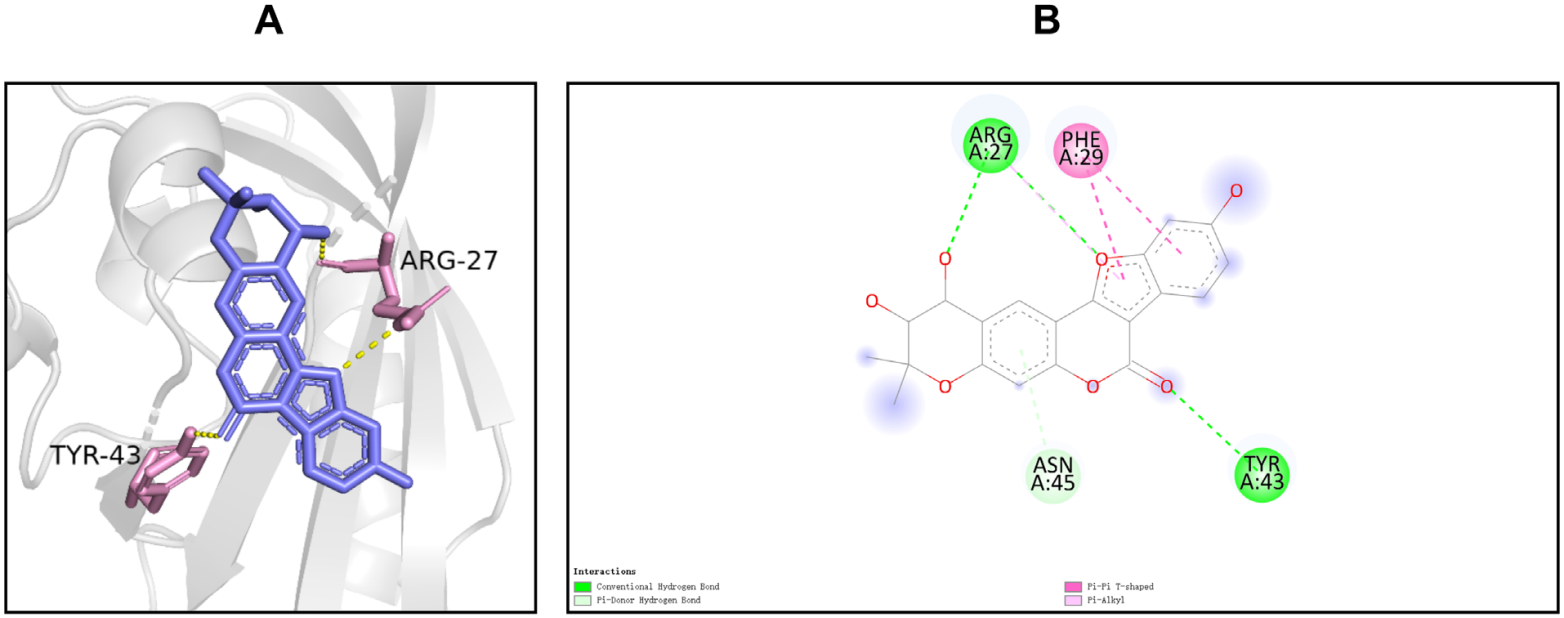
The binding mode of corylidin at the binding site of KCNH2. (A) The schematic diagram of the 3D mode. (B) Schematic diagram of the 2D mode. Through its Arg‐27 and Tyr‐43, KCNH2 formed a hydrogen bond with corylidin, while Asn‐45 and corylidin formed a weak hydrogen bond with the Pi bond as the donor; Phe‐29 and corylidin formed a T‐type Pi–Pi interaction, and Arg‐27 and corylidin formed a Pi‐alkyl interaction.

**FIGURE 8:**
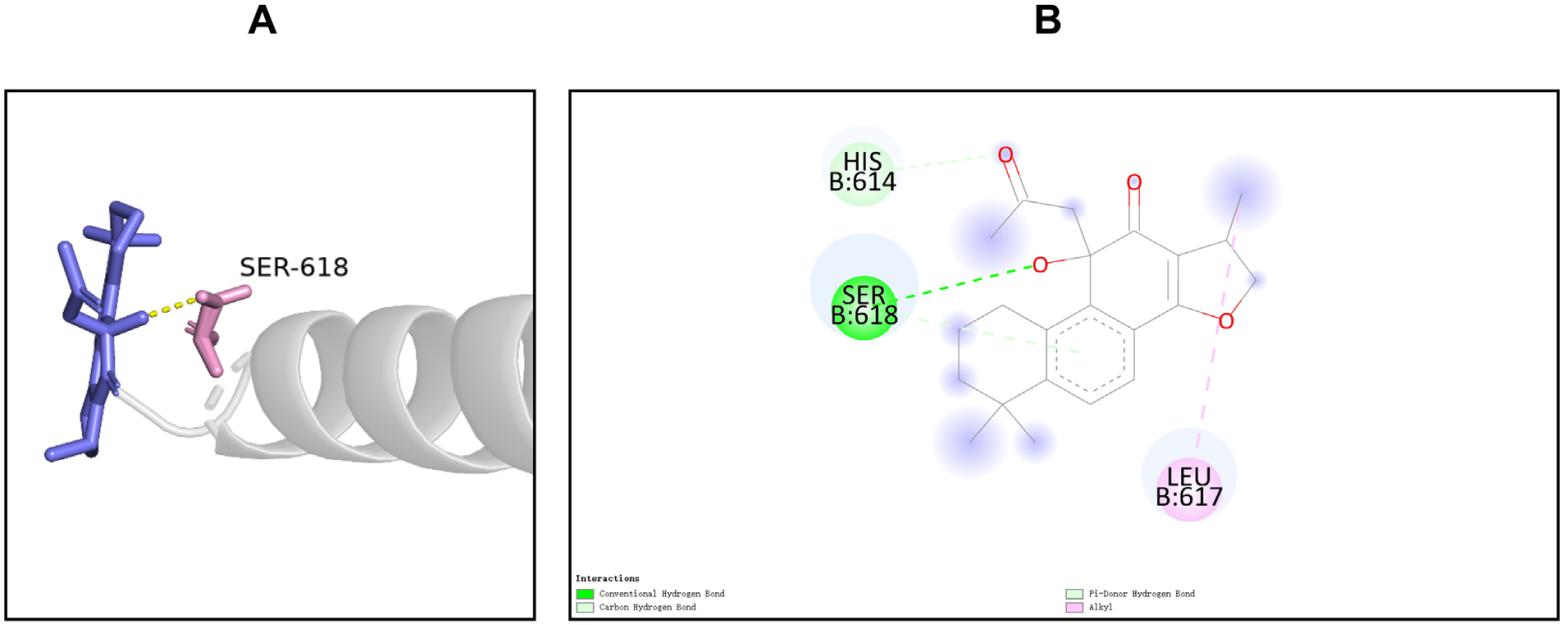
The binding mode of danshenol B at the binding site of KCNQ1. (A) The schematic diagram of the 3D mode. (B) The schematic diagram of the 2D mode. KCNQ1 formed a hydrogen bond with danshenol B through its Ser‐618. Meanwhile, Ser‐618 and danshenol B formed a weak hydrogen bond with Pi bond as the donor; His‐614 and danshenol B formed a C‐H interaction, while Leu‐627 and danshenol B formed an alkyl interaction.

**FIGURE 9:**
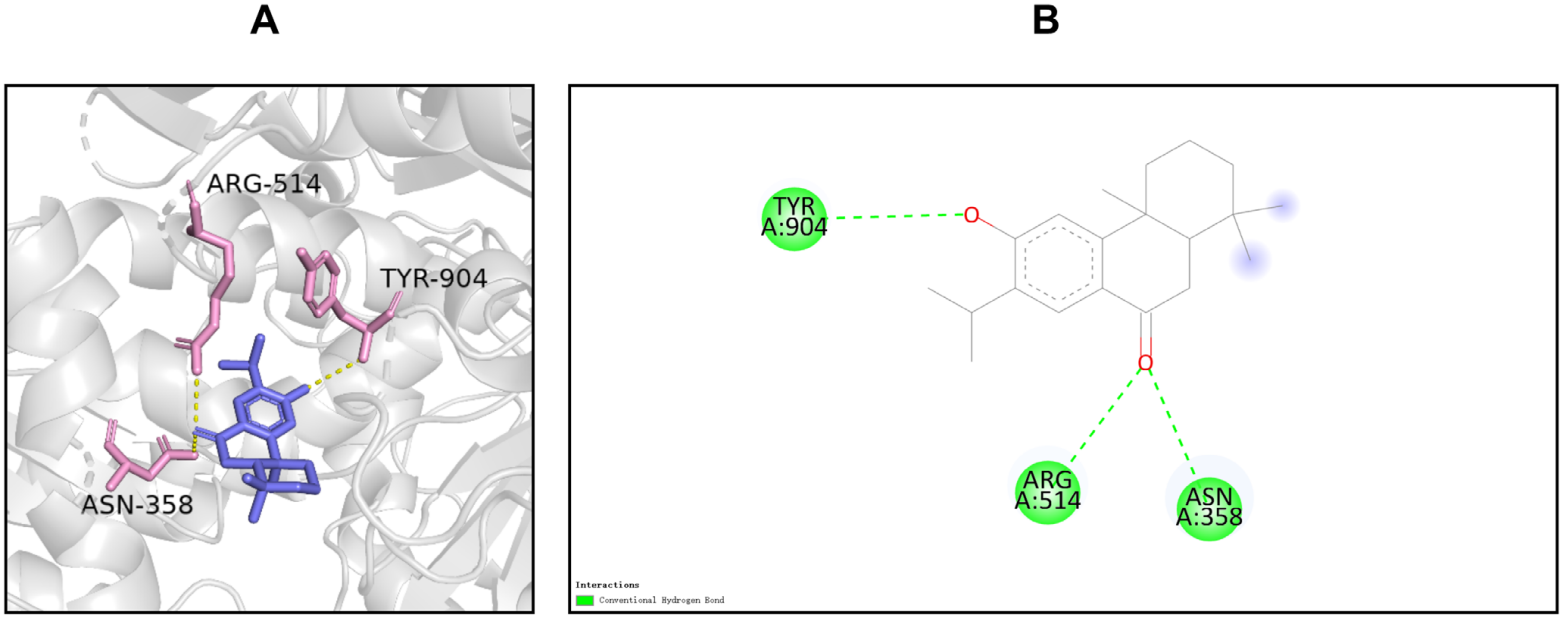
The binding mode of sugiol at the binding site of KCNMA1. (A) The schematic diagram of the 3D mode. (B) The schematic diagram of the 2D mode. KCNMA1 interacts with sugiol through its Tyr‐904, Arg‐514, and Asn‐358 to form hydrogen bonds.

The results of compound‐target network analysis revealed that 16 compounds of SXSM were closely related to the core targets BMP4, KCNH2, KCNQ1, and KCNMA1. To simulate the binding modes of these compound molecules and target proteins, molecular docking was performed (Table 4). The binding energy is shown in Table 5, and the parameter of molecular docking is shown in Table S2. Generally, binding energy < −5.0 kcal/mol is indicative of a good binding activity (Wang et al. 2023a, 2023b). In order to further explain the binding of small molecules to protein receptors, these molecules were sorted according to the minimum binding energy. Next, the small molecules with the best binding activity toward BMP4, KCNH2, KCNQ1, and KCNMA1 were selected. Particularly, this included erythrinin A and BMP4, corylidin and KCNH2, danshenol B and KCNQ1, and sugiol and KCNMA1. The interaction between the molecules and the target protein was further subjected to visual analysis (Figures 6, 7, 8, 9).

**TABLE 4:** The compounds that interact with BMP4, KCNH2, KCNQ1, and KCNMA1.

NO.	Name	Structure	Target
BGZ15	Bavachin	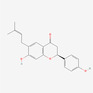	BMP4
BGZ28	Erythrinin A	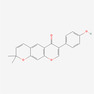	BMP4
XX1	4,9‐Dimethoxy‐1‐vinyl‐beta‐carboline	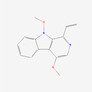	BMP4 KCNH2
HS	(E,E,E,E)‐Squalene	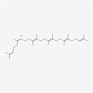	KCNH2
YYH11	8‐Prenyl‐flavone	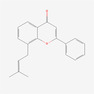	KCNH2
BGZ5	Corylidin	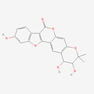	KCNH2
BGZ10	12,13‐Epoxybakuchiol	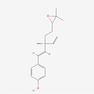	KCNH2
GQZ4	Atropine	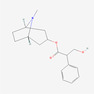	KCNH2
GQZ5	24‐Methylenelophenol	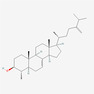	KCNH2
MH2	Squalene	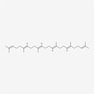	KCNH2
MH7 DS13	Eriodictyol	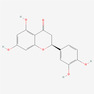	KCNH2
DS21	(1R)‐5‐hydroxy‐1,6,6‐trimethyl‐2,7,8,9‐tetrahydro‐1H‐naphtho[1,2‐g][1]benzofuran‐10,11‐dione	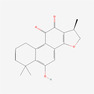	KCNH2
DS14	Danshenol A	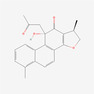	KCNQ1
DS15	Danshenol B	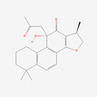	KCNQ1
DS6	Arucadiol	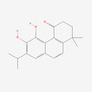	KCNMA1
DS28	Sugiol	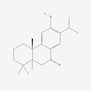	KCNMA1

**TABLE 5:** Analysis of the binding energy of 16 molecules with 4 target proteins.

Active ingredient	Target protein	The lowest binding energy (kcal/mol)
Bavachin	BMP4	−7.3
Erythrinin A	BMP4	−8
4,9‐Dimethoxy‐1‐vinyl‐beta‐carboline	BMP4	−6
4,9‐Dimethoxy‐1‐vinyl‐beta‐carboline	KCNH2	−5.2
(E,E,E,E)‐Squalene	KCNH2	−3.6
8‐Prenyl‐flavone	KCNH2	−6
Corylidin	KCNH2	−6.6
12,13‐Epoxybakuchiol	KCNH2	−4.8
Atropine	KCNH2	−5.3
24‐Methylenelophenol	KCNH2	−5.7
Squalene	KCNH2	−3.6
Eriodictyol	KCNH2	−5.7
(1R)‐5‐hydroxy‐1,6,6‐trimethyl‐2,7,8,9‐tetrahydro‐1H‐naphtho[1,2‐g][1]benzofuran‐10,11‐dione	KCNH2	−6
Danshenol A	KCNQ1	−6.1
Danshenol B	KCNQ1	−6.2
Arucadiol	KCNMA1	−7.7
Sugiol	KCNMA1	−7.9

### 3.6 Effect of SXSM on the Expression of *Bmp4*, *Tbx3*, and *Hcn4* in HL‐1 Cells

**FIGURE 10:**
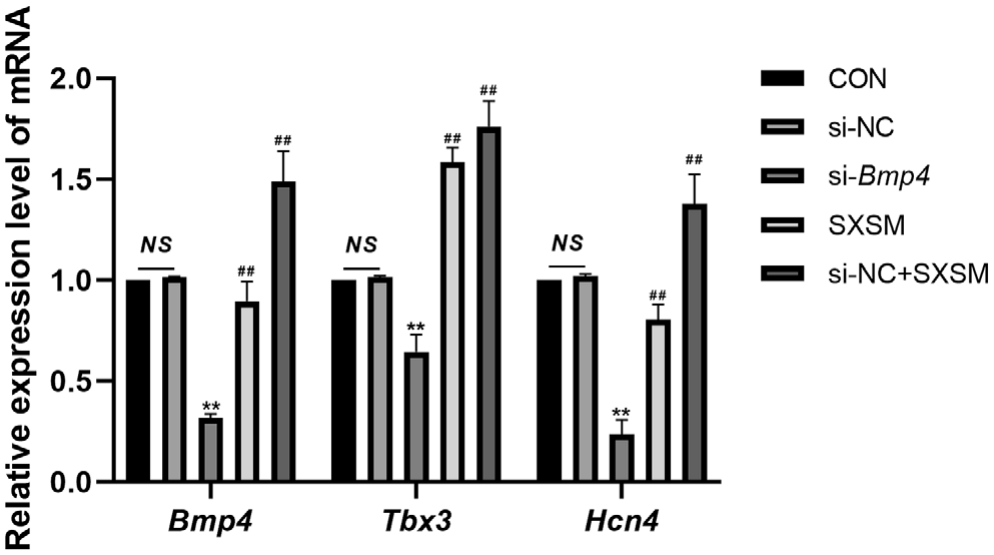
Expressions of mRNA of *Bmp4*, *Tbx3*, and *Hcn4* detected by RT‐qPCR. Comparison of the RT‐qPCR results. **p < 0.01, when compared with the si‐NC group. ^##^ *p* < 0.01, when compared with the si‐*Bmp4* group. *NS*, Not statistically significant.

To verify whether SXSM could regulate the *Bmp4*/*Tbx3*/*Hcn4* pathway, we used HL‐1 cells to simulate the P cells of SAN, which knocked down the expression of *Bmp4* with siRNA and treated with SXSM. The mRNA expressions of *Bmp4*, *Tbx3*, and *Hcn4* in HL‐1 cells in each group were determined by RT‐qPCR (Figure 10). The results revealed that, in comparison with the si‐NC group, the expressions of *Bmp4*, *Tbx3*, and *Hcn4* were reduced in the si‐*Bmp4* group (*p* < 0.01). When compared with the model group, the expressions of *Bmp4*, *Tbx3*, and *Hcn4* were increased in the SXSM group (*p* < 0.01).

### 3.7 Effect of SXSM on the Expression of *Kcnh2*, *Kcnq1*, and *Kcnma1* in HL‐1 Cells

**FIGURE 11:**
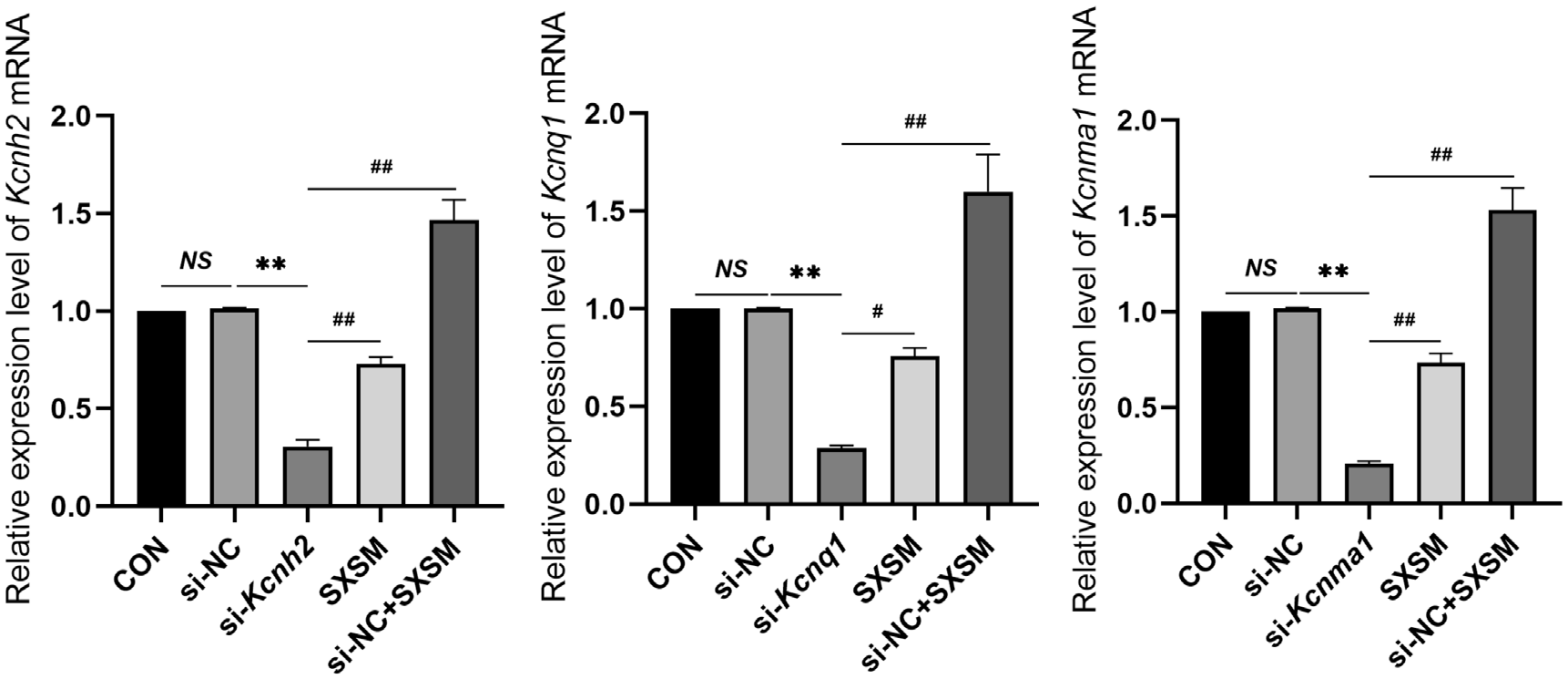
Expression of the mRNA of three genes detected by RT‐qPCR. Comparison of the RT‐qPCR results. **p < 0.01, ^#^ *p* < 0.05, ^##^ *p* < 0.01. *NS*, Not statistically significant.

To verify whether SXSM could regulate the expression of *Kcnh2*, *Kcnq1*, and *Kcnma1*, we specifically knocked down their mRNA levels in the HL‐1 cell line with siRNA and added SXSM. The expressions of *Kcnh2*, *Kcnq1*, and *Kcnma1* in HL‐1 cells in each group were determined by PT‐qPCR (Figure 11). The results showed that, in comparison with the si‐NC group, the expressions of *Kcnh2*, *Kcnq1*, and *Kcnma1* were reduced in the model group (*p* < 0.01). When compared with the model group, the expressions of *Kcnh2*, *Kcnq1*, and *Kcnma1* were increased in the SXSM group (*p* < 0.05, *p* < 0.01).

### 3.8 Effect of SXSM on the Beating Rates and FPD of hiPSC‐AMs

**FIGURE 12:**
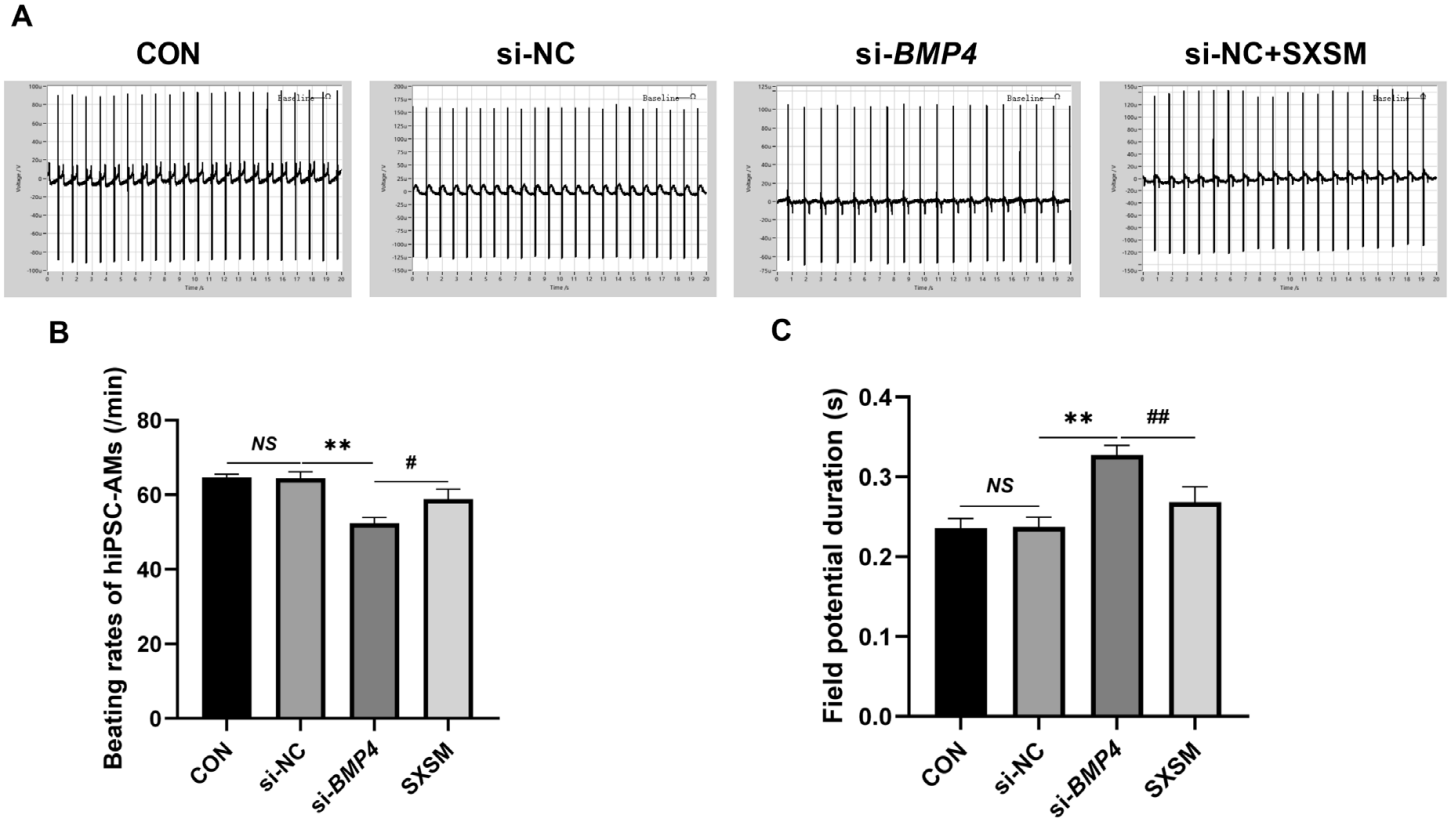
FPD and the beating rates in hiPSC‐AMs. (A) Representative field potential traces in hiPSC‐AMs. (B, C) Comparison of the beating rates and FPD in each group. **p < 0.01, ^#^ *p* < 0.05, ^##^ *p* < 0.01. *NS*, Not statistically significant.

**FIGURE 13:**
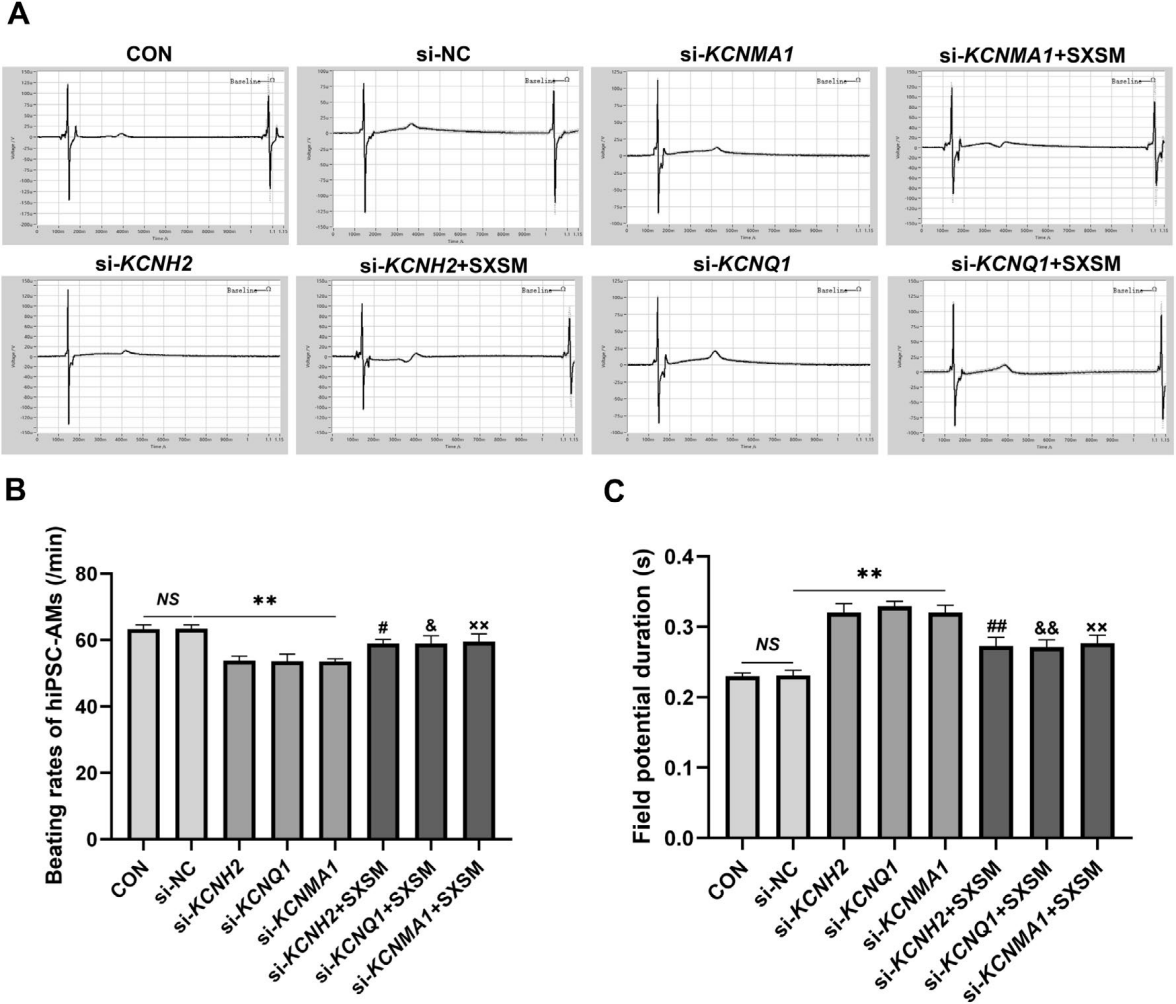
FPD and the beating rates in hiPSC‐AMs. (A) Representative field potential traces in hiPSC‐AMs. (B, C) Comparison of the beating rates and FPD in each group. **p < 0.01. ^#^ *p* < 0.05, ^##^ *p* < 0.01, when compared with the si‐*KCNH2* group. ^&^ *p* < 0.05, ^&&^ *p* < 0.01, when compared with the si‐*KCNQ1* group. ^××^ *p* < 0.01, when compared with the si‐*KCNMA1* group. *NS*, Not statistically significant.

We used autonomously beating hiPSC‐AMs to simulate the P cells of SAN, knocked down the expressions of *BMP4* by siRNA, and added SXSM. We then determined the beating rates and FPD of hiPSC‐AMs in each group. The electrophysiological assay results showed that, in comparison with the control group, the beating rates were reduced and the FPD was prolonged in the model group (Figure 12A–C; *p* < 0.01). After the addition of SXSM, the FPD was shortened and the beating rates were increased (Figure 12A–C; *p* < 0.05, *p* < 0.01). To verify whether SXSM could increase the beating rates of hiPSC‐AMs by regulating the expression of *KCNH2*, *KCNQI*, and *KCNMA1*, we specifically knocked down their mRNA levels in hiPSC‐AMs with siRNA and added SXSM. The results suggested that, in comparison with the control group, the beating rates were reduced and the FPD was prolonged in the model group (Figure 13A–C; *p* < 0.01). When compared with the model group, the FPD was shortened and the beating rates increased in the SXSM group (Figure 13A–C; *p* < 0.05, *p* < 0.01).

## 4 Discussion

The pathophysiology of SSS is varied and usually involves complex electrophysiological and structural remodeling. The sinoatrial node is comprised of a complex matrix of pacemaker cells, transitional cells, endothelial cells, fibroblasts, and extracellular scaffolding and is characterized by a unique ion channel and connexin expression profile that results in chronotropic automaticity. The underlying mechanism of SSS mostly involved the dysfunction of P cells in SAN (Liu and Hou 2023). The formation and maintenance of cardiac pacemaking functions are usually regulated by a series of specific genes. These genes regulate SAN‐specific ion channels, which play a fundamental role in regulating the action potential of P cells, thereby affecting the heart rate. The present study focused on SXSM, a Chinese patent medicine, which is known to exhibit remarkable curative effects and has a good safety profile for use in the treatment of SSS. This study discusses the underlying mechanism responsible for its therapeutic effect on SSS by using a combination of network pharmacology and in vitro experiments.

In this study, we screened 55 potential targets of SXSM for SSS through network pharmacology and analyzed the core mechanisms by GO and KEGG enrichment analyses. We observed that the targets of SXSM in the treatment of SSS were mostly concentrated in the function of the regulation of the membrane potential, while the second was the regulation of ion transmembrane transport, suggesting that these are the main mechanisms of SXSM involved in SSS treatment. The membrane potential of myocardial cells changes with the opening and closing of specific ion channels, which, in turn, generates the action potential duration (APD). The APD of P cells in SAN is categorized into the depolarization, repolarization, and spontaneous depolarization phases. When the spontaneous depolarization reaches the threshold membrane potential, it triggers a new action potential, such that rhythmic excitation is continuously generated. Therefore, the shorter the APD, the more rhythmic excitations P cells automatically generate, and the faster the heart rate. HCN4, CACNAIC, CACNA1H, CACNA2D1, KCNH2, KCNQ1, and KCNMA1 are the ion channels that play a major role in the regulation of APD (Hennis et al. 2022; Charpentier et al. 2010; Guo et al. 2011; Schmidt and Peyronnet 2018). Heart development is another important molecular mechanism, and BMP4 is the key target during the cardiomyocyte differentiation process (Wang et al. 2023a, 2023b). Therefore, we selected BMP4, KCNH2, KCNQ1, and KCNMA1 as the key targets in this study to explain the main mechanism of SXSM involved in the treatment of SSS.

During the development of the SAN, the genetic program for pacemaker cells is promoted while the genetic program that promotes chamber specification is inhibited; this process is tightly controlled by a series of transcription factors (Van Eif et al. 2018). In recent years, the role of bone morphogenetic protein (BMP) in animal embryo development and cell function has been widely explored (Chen, Zhao, and Mundy 2004). Among these, *Bmp4* is known to regulate the formation of P cells in SAN, which is adequate and necessary for the induction of the *Hcn4* expression (Hashem et al. 2013). BMP4 treatment promoted efficient differentiation into spontaneously beating embryoid bodies (EBs) in hiPSCs lines; these cells may develop into SAN (Kimura et al. 2019). It has been previously reported that the loss of function of *Bmp4* could lead to abnormal heart structure and even embryo death (Uchimura et al. 2009). Past studies have reported that *Bmp4* could reprogram cardiac myocytes into pacemaker‐like cells (Haraguchi et al. 2015). *Bmp4* was also considered a new strategy for the construction of biological pacemakers (Sun et al. 2015). It has previously been shown that *Bmp4* could bind to the promoter of transcription factor *Tbx3* and promote its expression. *Tbx3* is usually expressed in SAN, atrioventricular node (AVN), and the proximal part of His bundle. During the formation and maintenance of the cardiac pacemaking and conduction systems, it can inhibit the gene expression in myocardial working cells, thereby separating them from the cardiac chamber (Hoogaars et al. 2004; Wu et al. 2019). Further experiments revealed that *Tbx3* could reprogram terminally differentiated working cardiomyocytes, thereby facilitating biological pacemaker formation (Wiese et al. 2009; Bakker et al. 2012; Jensen et al. 2013; Zhao et al. 2020). *Tbx3* could activate the expression of *Hcn4* and other pacemaker cell‐specific genes. *Hcn4* is usually considered to be the molecular marker of the cardiac pacemaking system and serves as an important research target for biological pacemaking (Saito et al. 2015; Li et al. 2020). It has been previously shown that the *Hcn4* mutation could lead to SSS (Milanesi et al. 2006; Laish‐Farkash et al. 2010). Several clinical studies have reported that the loss of function of *Hcn4* could result in bradycardia (Hoogaars et al. 2007; Verkerk and Wilders 2015). Saito et al., for instance, transplanted mouse embryonic stem cell‐derived cardiomyocytes overexpressing *Hcn4* into the rat model for bradycardia and reported an increase in the heart rate (Saito et al. 2018). The HCN4 channel regulates the mixed Na^+^/K^+^ current, *I*_f_, which is referred to as the “pacemaker current,” and plays an important role in regulating neuronal excitability and cardiac autonomic rhythm (DiFrancesco 2010). The HCN4 channel is activated during the spontaneous depolarization phase of APD. When the HCN4 channel opens, the enhancement of the inward current leads to membrane depolarization. When the spontaneous depolarization reaches the potential threshold (−50 mv), the calcium channel on the cell membrane gets activated and a new APD is generated. Therefore, the more the Hcn4 channel is expressed, the more *I*_f_ current passes through the membrane, and higher is the autorhythmicity of P cells.

In order to verify whether SXSM could improve the SAN function by regulating the *Bmp4/Tbx3/Hcn4* pathway, HL‐1 cells were used to simulate P cells in vitro. The results of RT‐qPCR indicated that SXSM could increase the expression of *Bmp4*, transcription factor *Tbx3*, and ion channel *Hcn4* (*p* < 0.01). Because hiPSC‐AMs have a spontaneous beating rhythm, we used hiPSC‐AMs to simulate P cells of SAN to observe whether the promotion of SXSM on the expression of the *Bmp4/Tbx3/Hcn4* pathway affects the spontaneous beating rate. The knockdown of *BMP4* resulted in a reduction of the beating rates of hiPSC‐AMs, while the FPD was prolonged. After the addition of SXSM, the FPD was shortened and the beating rates were increased. We, therefore, speculated that SXSM may increase the expression of the downstream *Hcn4* channel by promoting the expression of the *Bmp4/Tbx3/Hcn4* pathway, thus increasing the *I*_f_ current density, shortening the APD, and increasing the heart rate.

The effect of the repolarization phase on the heart rate in the APD of P cells is also extremely important. The potassium channels play an important role in promoting P‐cell membrane repolarization during APD. KCNH2, KCNQ1, and KCNMA1 are the main ion channels involved in this process. The HERG channel encoded by *KCNH2* and the KvLQT1 channel encoded by *KCNQ1* are the main members of the α‐subunit of voltage‐gated potassium channels. Particularly, *KCNH2* is involved in the regulation of rapidly activating delayed rectifier potassium current *I*_Kr_ (Corponi et al. 2019). In comparison, *KCNQ1* regulates the slowly activating delayed rectifier potassium channel current, *I*_Ks_. In the repolarization phase of the action potential phase 3, the potassium channels are activated and opened, leading to potassium ion outflow. When the membrane potential reaches about −50 mV, the depolarization is re‐activated to complete an APD. Therefore, the more the potassium channels are expressed, the more potassium ion outflow in the repolarization phase, and the faster the spontaneous beating rates of P cells. Potassium channel blocker E‐4031 can decrease the pacemaking rate of SAN by reducing the maximum repolarization potential, which, in turn, affects the full activation of *I*_f_. It has previously been reported that mutations in *KCNH2* can cause SSS and long QT syndrome (LQTS) (Yang et al. 2022). There are two types of mutations in *KCNQ1*: Haploinsufficient *KCNQ1* mutations and dominant‐negative mutations. In these two mutations, the *I*_Ks_ reduction may range from mild (> 50%) to significant (< 93.75%) (Schwartz et al. 2021). Clinical studies have reported that the *KCNQ1* mutation can cause sinus bradycardia (Henrion et al. 2012; Ki et al. 2014). Furthermore, several studies have confirmed that *KCNQ1* dysfunction can cause SSS and other heart diseases, such as long QT interval syndrome and abnormal internode conduction (Demolombe et al. 2001; Hong et al. 2005; Zhou et al. 2019). *KCNMA1* encodes a large conductance voltage potassium channel that is calcium‐sensitive, which regulates the export of potassium ions and contributes to the repolarization of the membrane potential (Basrai et al. 2002). Past clinical studies have reported that the loss of function of this channel can slow down the heart rate, which further contributes to the development of SSS (Imlach et al. 2010; Lai et al. 2014; Pineda et al. 2021).

**FIGURE 14:**
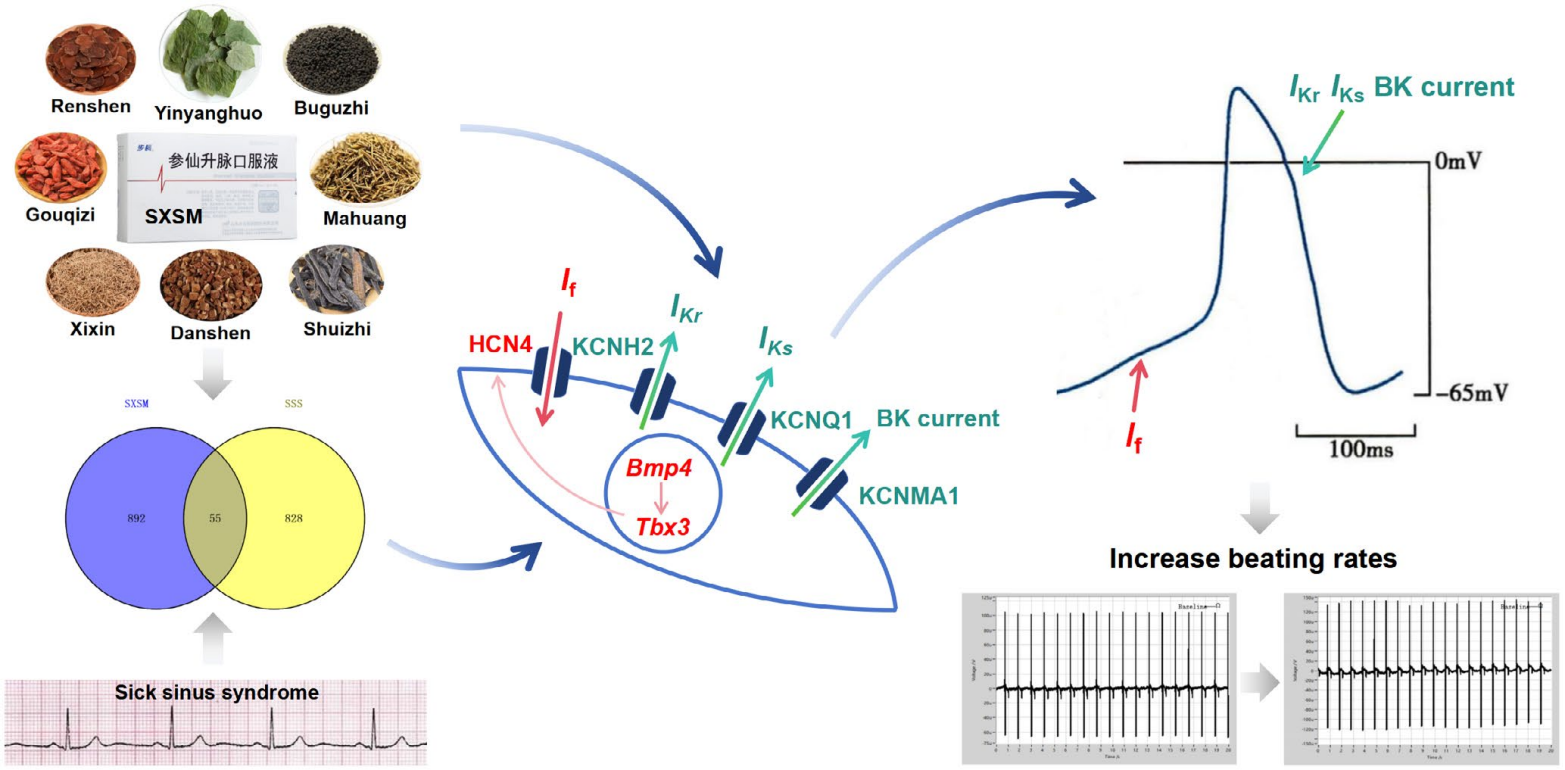
Summary of the main mechanism of SXSM in the treatment of SSS.

To verify whether SXSM could increase the heart rate by regulating these potassium ion channels, in the present study, the expression of *Kcnh2*, *Kcnq1*, and *Kcnma1* was knocked down in HL‐1 cells. Following this, SXSM was added, which increased the expression of *Kcnh2*, *Kcnq1*, and *Kcnma1* (*p* < 0.05, *p* < 0.01). Meanwhile, the expression of *KCNH2*, *KCNQ1*, and *KCNMA1* was knocked down in hiPSC‐AMs; the results indicated that a decrease in *KCNH2*, *KCNQ1*, and *KCNMA1* expression could prolong the FPD and lower the beating rates of hiPSC‐AMs, suggesting that decreasing the expression of *KCNH2*, *KCNQ1*, and *KCNMA1* channels may slow down the heart rate by prolonging APD. After the addition of SXSM, the FPD was shortened and the beating rates were increased. All these results indicated that SXSM may shorten the APD of P cells by increasing the expression of these pacemaking‐related potassium channels and increasing the heart rate (Figure 14).

Molecular docking results further provided a visual analysis of the interaction between 16 active compounds of SXSM and BMP4, KCNH2, KCNMA1, and KCNQ1. Most of these compounds have been reported in references (Xiang et al. 2018; Zong et al. 2024). The present results suggested that most compound molecules exhibited a good binding effect toward these four targets. In the future, we plan to isolate and purify these compounds to explore their therapeutic mechanisms for SSS toward increasing the efficiency and reducing the toxicity of SXSM.

## 5 Conclusion

SSS is a set of diseases with abnormal cardiac pacing; with the progression of this disease, it may lead to end‐organ hypoperfusion. In patients who are not candidates for pacemaker therapy, SXSM is the first Chinese patent medicine specially designed for SSS that has curative effects and low side effects. Our study analyzed the potential mechanisms of SXSM involved in the treatment of SSS based on network pharmacology prediction. We found that SXSM may increase the heart rate by regulating P cell gene programming and the transmembrane transport of potassium ions. Specifically, SXSM could regulate the *Bmp4/Tbx3/Hcn4* pathway and increase the expression of the *Hcn4* channel. Meanwhile, it increased the expression of three potassium channels, namely *Kcnh2*, *Kcnq1*, and *Kcnma1*. These regulations may shorten the APD of P cells and increase the heart rate.

## Author Contributions

**Ping Hou:** conceptualization (supporting), data curation (supporting), formal analysis (supporting), funding acquisition (lead), methodology (supporting), project administration (supporting), supervision (supporting), validation (supporting), writing – original draft (supporting), writing – review and editing (supporting). **Heng Zhang:** data curation (supporting), formal analysis (supporting), methodology (supporting), software (supporting). **Dong‐Yu Min:** formal analysis (supporting), methodology (supporting), software (supporting). **Jie Wu:** data curation (supporting), formal analysis (supporting). **Chen Chen:** data curation (supporting), formal analysis (supporting). **Jie Wang:** data curation (supporting), formal analysis (supporting). **Yong‐Ping Lu:** data curation (supporting), software (supporting). **Ying‐Jia Yao:** data curation (supporting), software (supporting). **Ling‐Kang Li:** data curation (supporting). **Yue Liu:** conceptualization (lead), data curation (lead), formal analysis (lead), funding acquisition (supporting), methodology (lead), project administration (lead), software (lead), supervision (lead), writing – original draft (lead), writing – review and editing (lead).

## Conflicts of Interest

The authors declare no conflicts of interest.

## Supporting information


**Table S1.** Information of target proteins for molecular docking.

**Table S2.** The parameter and result of molecular docking.

## Data Availability

The data that support the findings of this study are available on request from the corresponding author.
