# Supplementary material for: Evaluation of the Potential Targets of Shenxian–Shengmai Oral Liquid in Treating Sick Sinus Syndrome Based on Network Pharmacology and Molecular Docking
Source: Food Sci Nutr. 2024 Nov 12;12(12):10517–34. doi: 10.1002/fsn3.4587 (PMC11666830; doi:10.1002/fsn3.4587)
Supplement: Supplementary file 1 — Table S1. Information of target proteins for molecular docking. [file FSN3-12-10517-s002.docx]

**TABLE S1. Information of target proteins for molecular docking.**

| Protein | PDB ID | Organism | Resolution | Co-crystallized ligand |
| --- | --- | --- | --- | --- |
| KCNH2 | 1BYW | Homo sapiens | 2.60 Å | None |
| KCNQ1 | 3BJ4 | Homo sapiens | 2.00 Å | Nickel (II) ion |
| KCNMA1 | 3MT5 | Homo sapiens | 3.00 Å | Sulfate ion |
| BMP4 | - | Homo sapiens | - | - |
