# Supplementary material for: Evaluation of the Potential Targets of Shenxian–Shengmai Oral Liquid in Treating Sick Sinus Syndrome Based on Network Pharmacology and Molecular Docking
Source: Food Sci Nutr. 2024 Nov 12;12(12):10517–34. doi: 10.1002/fsn3.4587 (PMC11666830; doi:10.1002/fsn3.4587)
Supplement: Supplementary file 2 — Table S2. The parameter and result of molecular docking. [file FSN3-12-10517-s001.docx]

**TABLE S2. The parameter and result of molecular docking.**

| Molecule name | Potential target | Center of Grid Box (Å) | Size of Grid Box (Å) | Binding free energies (kcal/mol) |
| --- | --- | --- | --- | --- |
| Bavachin | BMP4 | -4.3, 1.6, 5.9 | 79.9×75.2×99.9 | -7.3 |
| Erythrinin A | BMP4 | -4.3, 1.6, 5.9 | 79.9×75.2×99.9 | -8 |
| 4,9-Dimethoxy-1-vinyl-beta-carboline | BMP4 | -4.3, 1.6, 5.9 | 79.9×75.2×99.9 | -6 |
| 4,9-Dimethoxy-1-vinyl-beta-carboline | KCNH2 | 11.5, 14.4, 12.7 | 47.2×40×45.1 | -5.2 |
| (E,E,E,E)-Squalene | KCNH2 | 11.5, 14.4, 12.7 | 47.2×40×45.1 | -3.6 |
| 8-Prenyl-flavone | KCNH2 | 11.5, 14.4, 12.7 | 47.2×40×45.1 | -6 |
| Corylidin | KCNH2 | 11.5, 14.4, 12.7 | 47.2×40×45.1 | -6.6 |
| 12,13-Epoxybakuchiol | KCNH2 | 11.5, 14.4, 12.7 | 47.2×40×45.1 | -4.8 |
| Atropine | KCNH2 | 11.5, 14.4, 12.7 | 47.2×40×45.1 | -5.3 |
| 24-Methylenelophenol | KCNH2 | 11.5, 14.4, 12.7 | 47.2×40×45.1 | -5.7 |
| Squalene | KCNH2 | 11.5, 14.4, 12.7 | 47.2×40×45.1 | -3.6 |
| Eriodictyol | KCNH2 | 11.5, 14.4, 12.7 | 47.2×40×45.1 | -5.7 |
| (1R)-5-hydroxy-1,6,6-  trimethyl-2,7,8,9-  tetrahydro-1H-naphtho[1,2-g][1]benzofuran -10,11-dione | KCNH2 | 11.5, 14.4, 12.7 | 47.2×40×45.1 | -6 |
| Danshenol A | KCNQ1 | -1.7, -6.1, 4.4 | 31.6×65.5×26.6 | -6.1 |
| Danshenol B | KCNQ1 | -1.7, -6.1, 4.4 | 31.6×65.5×26.6 | -6.2 |
| Arucadiol | KCNMA1 | 36.8, 38, 20.9 | 80×78.4×90.2 | -7.7 |
| Sugiol | KCNMA1 | 36.8, 38, 20.9 | 80×78.4×90.2 | -7.9 |
